# Predictors of Academic Adjustment Among International Students in Rural Southern USA

**DOI:** 10.3390/ijerph22020253

**Published:** 2025-02-11

**Authors:** Ruaa Al Juboori, Dylan Barker, Yi Jin Kim

**Affiliations:** 1 Department of Public Health, School of Applied Sciences, University of Mississippi, Oxford, MS 38677, USA; dabarke1@go.olemiss.edu; 2 Institute of Child Nutrition (ICN), School of Applied Sciences, University of Mississippi, Oxford, MS 38677, USA; 3 Department of Social Work, School of Applied Sciences, University of Mississippi, Oxford, MS 38677, USA; yjkim@olemiss.edu

**Keywords:** rural higher education, mental well-being, social support, academic adaptation, international students, cultural integration

## Abstract

Academic adaptation is crucial for the success and well-being of international students, especially in rural areas where cultural and social support resources may be limited. This study aimed to identify predictors of academic adaptation among international students in the rural southern United States, focusing on social support, cultural integration, alcohol use, and acculturative stress. A cross-sectional study was conducted with 141 international students from two universities: the University of Mississippi/Oxford campus and the University of Alabama. Data were collected through a web-based survey using validated scales to measure cultural integration, acculturative stress, social support, alcohol use, and academic adaptation. Pearson correlation and multiple linear regression analyses were performed to identify significant predictors of academic adaptation. The sample consisted of 54.6% male and 45.4% female students, with a mean age of 29.4 years. The majority were graduate students (89.4%), with the majority being funded by graduate teaching or research assistantships (78.7%). Correlation analyses showed that social support was positively correlated with academic adaptation (r = 0.62, *p* < 0.01). Also, cultural integration was positively corelated with academic adaptation (r = 0.33, *p* < 0.001). However, acculturative stress was negatively correlated with academic adaptation (r = −0.20, *p* < 0.05). The fully adjusted regression analyses identified social support as a positive predictor of academic adaptation among international students in rural U.S. universities. The model demonstrated that each unit increase in the social support score was associated with a 0.61 increase in the academic adaptation score (95% CI [0.44, 0.79], *p* < 0.001). These findings highlight the need for tailored support programs addressing the unique challenges faced by this population to enhance their academic success and overall well-being.

## 1. Introduction

### 1.1. Background

International students play an important role in enriching the cultural diversity and academic environment of universities worldwide. In recent years, the presence of international students in U.S. higher education institutions has grown significantly. For the 2022/2023 academic year, the total number of international students at U.S. colleges and universities surged by 12% to 1,057,188, surpassing pre-pandemic levels and marking a strong recovery from the disruptions caused by the COVID-19 pandemic [1]. This resurgence is further reflected in the 14% increase in new international student enrollments, totaling 298,523, which builds on an 80% increase from the previous year. This growth spans all academic levels and fields of study, which highlight the continuing appeal of U.S. education to students from around the world [2]. China and India remain the leading countries of origin for international students. Together, they comprise 27% and 25% of the international student population, respectively. This accounts for over half (53%) of all international students in the U.S. Other significant contributors include South Korea, Canada, Brazil, Nigeria, Saudi Arabia, Japan, Taiwan, and Vietnam [2].

As international students increasingly enroll in U.S. institutions, they contribute to a richer learning environment for all students. While they bring diverse perspectives, they also face distinct challenges that can impact their academic adaptation [3,4]. This is a critical factor for their success and well-being in foreign academic environments. Academic adaptation refers to the process by which international students adjust to the academic norms and expectations of their new educational institutions [5]. Effective adaptation is crucial as it directly influences academic performance, retention rates, and overall student satisfaction [6].

The significance of proper academic adaptation among international students is highlighted by the challenges they encounter, including acculturative stress, social isolation, and differing academic expectations [7,8,9,10]. These challenges might be deepened in rural U.S. settings, where resources and social support systems are typically less prevalent compared to urban areas [11,12]. A lack of cultural integration and limited social support in these environments can exacerbate students’ adaptation difficulties.

Therefore, this study focuses on four specific predictors of academic adaptation including social support, cultural integration, alcohol use, and acculturative stress. Each of these factors has been selected based on their implications for the academic and psychological well-being of international students. First, social support is crucial in the adaptation process of international students. It provides protection against the psychological stresses of acculturation and can impact academic success [13,14,15]. More specifically, social networks, whether consisting of peers, family back home, or university staff, offer emotional, informational, and appraisal support that can ease the transition into a new academic and cultural environment [13,14,15]. This support is especially vital in rural areas, where international students might face greater isolation due to smaller, less diverse communities.

Second, cultural integration, the extent to which international students engage with and adapt to the host culture, affects their social interactions and their academic success [16,17,18]. Students who actively participate in their new cultural settings are likely to develop a better understanding of academic expectations and social norms, thereby easing their academic adaptation. This integration can be done through structured university programs, community engagement, and informal social interactions, which can be limited in rural settings [19]. Third, acculturative stress arises from the process of adapting to a new cultural environment and is a significant predictor of mental health issues among international students [7]. This stress can stem from language barriers, educational differences, and a lack of familiar social support structures. Therefore, it might negatively impact both mental health and academic performance. In rural areas, where cultural differences may be more pronounced and support services less accessible, acculturative stress can be particularly challenging [3].

Fourth, alcohol use is a prevalent issue among college students and warrants specific attention in the context of international students who might use alcohol as a coping mechanism for acculturative stress [20]. The relationship between stress and alcohol use is well-documented, with studies indicating that higher levels of stress can lead to increased alcohol consumption, which in turn can adversely affect academic performance and health [16,17,18]. Therefore, understanding this relationship is crucial for developing interventions that address healthy coping strategies.

Despite the established importance of these factors, research specifically addressing their association with academic adaptation among international students in rural U.S. contexts remains sparse. For example, Lee et al. (2013) found that rural international students experienced unique challenges related to cultural integration and alcohol use that were different from those experienced by their urban counterparts [21]. Similarly, Dixon et al. (2016) noted the need for more research on social support and its impact on alcohol use among international students in rural areas [22].

Therefore, the objective of this study is to investigate the association between social support, cultural integration, acculturative stress, and alcohol use, on the academic adaptation of international students in rural U.S. universities. This research aims to identify specific challenges and predictors of success within this context to inform targeted interventions that enhance academic outcomes and well-being for this population.

### 1.2. Research Questions and Hypotheses

1. How is social support associated with academic adaptation among international students in rural U.S. universities?

**H1.**  *Higher levels of social support are positively associated with academic adaptation*.

2. How is cultural integration associated with academic adaptation?

**H2.**  *Greater cultural integration is associated with higher academic adaptation*.

3. How is acculturative stress associated with academic adaptation?

**H3.**  *Acculturative stress is negatively associated with academic adaptation*.

4. How is alcohol use associated with academic adaptation?

**H4.**  *Increased alcohol use is negatively associated with academic adaptation*.

## 2. Materials and Methods

### 2.1. Study Design and Data Collection

This study used a cross-sectional design to examine the relationships between social support, cultural integration, acculturative stress, alcohol consumption, and academic adaptation among international students. The study respondents were 141 international students attending two southern rural universities (the University of Mississippi/Oxford and the University of Alabama). Participants were recruited through university mailing lists and international student offices. Eligibility criteria included being an international student aged 21 years or older and currently enrolled at one of the participating universities. Participation was voluntary, and all respondents provided informed digital consent prior to completing the survey. To maintain participant confidentiality, no identifying information was collected. However, participants were given the option to provide their school email address at the end of the survey to enter a raffle for one of 120 Amazon gift cards worth USD 25. This email information was used exclusively for contacting raffle winners. The raffle was conducted after data collection was completed, and winners were notified via their provided school email addresses.

Data were collected via an online survey administered through the Qualtrics platform during the 2022/2023 academic year. In this study, the target population consisted of international students at the University of Mississippi/Oxford and the University of Alabama. According to available data, the University of Mississippi hosts a total of 669 international students, while the University of Alabama has 1176 international students [23,24]. Our sample included 141 international students, with 65 participants from the University of Mississippi and 76 from the University of Alabama. This constitutes about 9.7% of the international student population at the University of Mississippi/Oxford and approximately 6.5% at the University of Alabama.

The survey included questions about the students’ age, gender, relationship status, academic level, funding source, length of stay in the U.S., and the world region from which the students came. Validated scales were used to measure social support [25], acculturative stress [26], cultural integration [27], academic adaptation [28], and alcohol use [29]. Supplementary Materials File S1 shows the detailed questions and the response options for the validated scales used in this study. The Academic Adaptation Scale was adopted from the General Adaptation Scale for International Students developed and validated by Polat et al. [30]. It measures students’ satisfaction and comfort with their academic environment. It consists of six items that inquire about aspects of academic experience, including satisfaction with academic progress, support received from teachers, assistance from classmates, comfort with teaching styles, collaboration on school projects, and overall support from the university. Respondents rate their agreement with each statement on a five-point Likert scale from Strongly Disagree to Strongly Agree. The validity and reliability for this scale was reported in previous study [30]. The mean score of this scale was calculated and included as a continuous variable in the model, with a Cronbach’s alpha of 0.87 obtained from our sample for this scale. A higher score indicates greater satisfaction and comfort within the academic environment.

The Alcohol Use Disorders Identification Test (AUDIT), a globally recognized screening tool designed to identify individuals at risk of alcohol-related problems, was used in this study. The AUDIT comprises ten questions that assess alcohol consumption, drinking behavior, and alcohol-related issues. Each question has a multi-point scale for responses, which are scored to quantify the level of risk [31,32]. The mean score of this scale was calculated and included as a continuous variable in the model. Previous studies have shown adequate reliability and validity of the AUDIT [31,32]. The Cronbach’s alpha from our sample was 0.73 for the AUDIT. Higher scores indicated greater risk.

The Multidimensional Scale of Perceived Social Support was used to measure social support in this study [33]. The Multidimensional Scale of Perceived Social Support is a survey instrument that measures the levels of social support an individual perceives from different sources, such as family, friends, and specific supportive figures or organizations. Respondents read 12 statements about the support they receive and express their level of agreement using a five-point Likert scale ranging from Strongly Disagree to Strongly Agree. The scale showed high validity and reliability [34,35]. The mean score of this scale was calculated and included as a continuous variable in the model, with a Cronbach’s alpha of 0.86 obtained from our sample for this scale. Higher scores reflected perceived higher support levels.

The Acculturative Stress Scale, which assesses the emotional and psychological challenges faced by individuals as they adapt to a new cultural environment, was used in this study [26]. This scale includes a series of 20 statements that measure feelings of homesickness, sadness due to unfamiliar surroundings, and loss concerning leaving family and friends behind. It also captures experiences of alienation, such as perceived hostility or discrimination from others and feelings of insecurity or fear due to cultural differences. Respondents indicate their level of agreement with each of the statements using a five-point Likert scale, ranging from Strongly Disagree to Strongly Agree. Previous studies showed high levels of validity and reliability for this scale [26,36,37,38]. The mean score of this scale was calculated and included as a continuous variable in the model, with a Cronbach’s alpha of 0.92 obtained from our sample for this scale. Higher scores indicated greater acculturative stress experienced.

Cultural integration comprised of ten mainstream items used by the Vancouver Index of Acculturation (VIA) [27,39]. The questions evaluated various aspects of engagement with and acceptance of American culture, such as participation in mainstream traditions, willingness to marry an American, enjoyment of social activities with Americans, and comfort with typical American interactions. It also measures the affinity for American entertainment, behavioral conformity to American norms, the importance placed on adopting American cultural practices, belief in American values, enjoyment of American humor, and interest in forming friendships with Americans. Respondents express their level of agreement with each statement using a five-point Likert scale ranging from Strongly Disagree to Strongly Agree. Previous studies showed high levels of validity and reliability for this scale [27,39]. The mean score of this scale was calculated and included as a continuous variable in the model, with a Cronbach’s alpha of 0.85 obtained from our sample for this scale. Higher scores indicated deeper integration into the host culture. The survey was designed to be completed in approximately 10 to 15 min.

### 2.2. Ethical Considerations

This study was approved by the University of Mississippi’s Institutional Review Board (IRB Protocol #24x-050). Participation was voluntary, and respondents could withdraw at any time without penalty. The survey included questions about potentially sensitive topics, such as psychological well-being and alcohol consumption. Participants were advised to contact their university counseling services if they experienced any distress while completing the survey.

### 2.3. Data Analysis

Descriptive statistics were used to summarize the demographic characteristics of the participants, as well as their responses to academic adaptation, cultural integration, acculturative stress, social support, and alcohol consumption scales. Pearson correlation analyses were conducted to examine the relationships among these variables.

To identify the variables associated with academic adaptation among international students, a progressive multiple regression analysis was conducted. The dependent variable was academic adaptation, measured through a validated scale in the survey. Independent variables included demographic factors (age, gender, relationship status, academic level, funding source, world region, and length of stay in the U.S.), social support, acculturative stress, cultural integration, and alcohol consumption.

The regression model was constructed in two stages. First, demographic and background variables were entered to control for their potential association with the outcome. In the second stage, social support, acculturative stress, cultural integration, and alcohol consumption were added to assess their association with academic adaptation while controlling for the demographic and the background variables. Variance inflation factors (VIF) were calculated to assess multicollinearity among predictors. The regression coefficients (B), 95% confidence intervals, and *p*-values, were calculated. All analyses were conducted using IBM SPSS Statistics (version 24), with statistical significance set at *p* < 0.05.

Prior to regression analysis, the normality of data was assessed. The results indicated that the data were normally distributed for the purposes of multiple regression. This analysis was not guided by a pre-established conceptual framework but was instead structured around the study’s objectives to explore the factors associated with academic adaptation among international students.

## 3. Results

### 3.1. Characteristics of the Study Sample

The study included a total of 141 participants. The gender distribution showed 54.6% (n = 77) identifying as male and 45.4% (n = 64) as female. Regarding the length of stay in the United States, around one third of participants (35.5%, n = 50) had been in the country for less than 12 months, followed by 29.8% (n = 42) who had stayed between 25 and 60 months, 23.4% (n = 33) who had stayed between 12 to 24 months, and 11.3% (n = 16) who had been in the U.S. for more than 60 months.

In terms of relationship status, over half of the participants were single (51.8%, n = 73), with 27.0% (n = 38) being married and 21.3% (n = 30) in a dating relationship. The majority of the sample were graduate students (89.4%, n = 126), while the remaining 10.6% (n = 15) were undergraduate students. Regarding the source of funding for their studies, 78.7% (n = 111) were supported by graduate teaching or research assistantships, and 21.3% (n = 30) relied on scholarships or family funds. The participants represented various world regions, with the highest percentage from the Southeast Asia Region (32.6%, n = 46), followed by the African Region (20.6%, n = 29), Eastern Mediterranean Region (17%, n = 24), Western Pacific Region (9.9%, n = 14), Region of the Americas (11.3%, n = 16), and European Region (8.5%, n = 12). The mean scores for social support, acculturative stress, cultural integration, academic adaptation, and alcohol use were 3.80 (SD = 0.74), 2.47 (SD = 0.78), 3.60 (SD = 0.70), 3.90 (SD = 0.88), and 2.11 (SD = 0.38), respectively. Please refer to Table 1 for a detailed description of the participant characteristics.

### 3.2. Correlation Analysis

Pearson correlation analyses were conducted to examine the relationships among social support, acculturative stress, cultural integration, alcohol consumption, and academic adaptation. Social support was found to be significantly positively correlated with academic adaptation (r = 0.62, *p* < 0.01). A significant negative correlation was observed between acculturative stress and academic adaptation (r = −0.20, *p* < 0.05). These correlations suggest that higher levels of social support are associated with better academic adaptation and cultural integration among the participants. Additionally, higher acculturative stress is associated with poorer academic adaptation. However, alcohol consumption did not show significant relationships with the other variables studied (see Table 2).

### 3.3. Regression Analysis

Regression analyses revealed that certain demographic and psychosocial factors are significant predictors of academic adaptation among international students. In the initial regression model, being married compared to being single was associated with an increase in the academic adaptation score of 0.37 (95% CI [0.02, 0.73], *p* = 0.04). Furthermore, the fully adjusted model demonstrated that each unit increase in the social support score was associated with a 0.61 increase in the academic adaptation score (95% CI [0.44, 0.79], *p* < 0.001). Other factors, such as age, gender, academic level, and alcohol consumption, did not show a significant relationship with academic adaptation. The coefficient of determination (R²) was 45% and 52% for Model 1 and model 2, respectively. It indicates that model 2 explains over half of the variability in academic adaptation scores. (See Table 3)

## 4. Discussion

In this study, we investigated how social support, cultural integration, alcohol use, and acculturative stress were associated with academic adaptation among international students in a rural Southern region of the United States. Our bivariate analyses suggested that social support and cultural integration were positively associated with academic adaptation. In contrast, higher levels of acculturative stress showed a negative relationship with academic adaptation. However, these associations were non-significant in the multivariate regression analyses except social support. These findings add to the body of research on international students’ experiences by clarifying which factors are associated with higher or lower likelihood of academic success in a rural context.

A key result was the significant association between social support and academic adaptation. This reinforces prior work highlighting the importance of supportive peer networks, family connections, and community resources for academic success among international students [8,21,40]. In rural regions, where international students may have fewer cultural or linguistic resources, peer and faculty support can take on even greater significance [21,22]. The significant relationship between social support and academic outcomes highlights the need for universities and community organizations to foster mentorship programs and inclusive events that encourage meaningful student interactions.

Our bivariate analyses also demonstrate that cultural integration plays a moderate significant role in promoting academic adaptation. Yet, this association was not significant in the multivariate regression model. Students who are actively engaged with local traditions, social norms, and community activities might be more comfortable navigating academic requirements. This aligns with recent research suggesting that cultural integration can help reduce sociocultural barriers and facilitate academic persistence [41,42,43]. For higher education institutions in rural settings, providing structured opportunities for cross-cultural engagement such as cultural fairs, exchange programs, or community-based projects may boost academic adaptation by helping students develop a sense of belonging. However, future studies might provide a better explanation of how the role of cultural integration might be diminished with the presence of other stressors like acculturative stress.

Regarding alcohol use, it showed no association with academic adaptation. This finding is opposite to what previous studies showed. For example, some studies showed that alcohol use was associated with negative academic achievements both among domestic and international students [44,45,46]. Previous studies suggested that problematic alcohol use may reflect attempts to cope with limited peer support or feelings of isolation [47,48]. Further research could clarify whether alcohol use mediates or moderates the relationship between social isolation and academic performance.

Similarly, acculturative stress demonstrated a negative weak correlation with academic adaptation, but the association was not significant in the multivariate regression model. This suggests that while academic and cultural demands can be overwhelming, international students with adequate support systems and opportunities for meaningful cultural integration are better able to cope [8]. Interventions that target stress reduction including counseling services, peer support networks, and international student clubs may be key in sustaining academic motivation and success in rural universities. Future studies could explore whether acculturative stress has distinct patterns in rural environments compared to urban settings, where services and diverse communities might be more readily available.

This study has several limitations that should be considered when interpreting the results. The cross-sectional design limits the ability to draw causal inferences, and the self-reported nature of the data may be subject to social desirability bias. Additionally, the study sample was drawn from only two universities in the rural southern U.S., which may limit the generalizability of the findings to other regions or types of institutions. Future research could build on these findings by exploring the longitudinal effects of social support, marital status, and acculturative stress on academic adaptation, as well as by examining these relationships in different geographic and institutional contexts.

## 5. Conclusions

This study highlights the importance of social support in promoting academic adaptation among international students in the rural southern U.S. The findings suggest that universities could prioritize the development of support systems that enhance social integration and provide targeted assistance to students who may be at greater risk of academic challenges. By doing so, universities could promote the success and well-being of their international student populations.

## Figures and Tables

**Table 1:** Characteristics of the study sample, N = 141.

Characteristics		Frequency	%
Gender	Female	64	45.4%
	Male	77	54.6%
Length of stay in the US	Less than 12 months (<1 year)	50	35.5%
	12–24 months (1 year–2 years)	33	23.4%
	25–60 months (>2 years–5 years)	42	29.8%
	More than 60 months (>5 years)	16	11.3%
Relationship status	Dating	30	21.3%
	Married	38	27%
	Single	73	51.8%
Academic level	Graduate	126	89.4%
	Undergraduate	15	10.6%
Funding source	Graduate teaching/research assistantship	111	78.7%
	Scholarship/Family funds	30	21.3%
World region	African Region	29	20.6%
	Eastern Mediterranean Region	24	17%
	European Region	12	8.5%
	Region of the Americas	16	11.3%
	Southeast Asia Region	46	32.6%
	Western Pacific Region	14	9.9%
University	The University of Mississippi/Oxford	65	46.1%
	University of Alabama	76	53.9%
		**Mean**	**SD**
Age		29.4	6.3
Social Support		3.80	0.74
Acculturative stress		2.47	0.78
Cultural Integration		3.60	0.70
Academic Adaptation		3.90	0.88
Alcohol Use		2.11	0.38

**Table 2:** Pearson correlation among the study scales, N = 141.

	Social Support	Acculturative Stress	Cultural Integration	Alcohol Consumption
Academic adaptation	0.62 **	−0.20 *	0.33 **	−0.06
Social support		−0.11	0.40 **	−0.03
Acculturative stress			−0.09	0.03
Cultural integration				0.14

** Correlation is significant at the 0.01 level (2-tailed), *. Correlation is significant at the 0.05 level (2-tailed).

**Table 3:** Regression analyses predicting academic adaptation, N = 141.

	Model 1					Model 2				
	95% CI					95% CI				
	*B*	*p* Value	Lower	Upper	VIF	*B*	*p* Value	Lower	Upper	VIF
Age	−0.02	0.12	−0.05	0.01	1.53	−0.01	0.61	−0.03	0.02	1.71
Gender (Ref = Male)										
Female	0.17	0.27	−0.13	0.46	1.11	0.06	0.61	−0.18	0.30	1.17
Relationship status (Ref = Single)										
Dating	0.28	0.16	−0.11	0.67	1.29	0.10	0.53	−0.23	0.43	1.46
Married	**0.37**	**0.04**	**0.02**	**0.73**	1.31	0.20	0.19	−0.10	0.50	1.40
Academic level (Ref = Undergraduate)										
Graduate	0.29	0.40	−0.38	0.95	2.19	0.31	0.26	−0.23	0.84	2.20
Funding source (Ref = Scholarship/family funds)										
Graduate teaching/research assistantship	−0.40	0.10	−0.88	0.07	1.96	−0.27	0.17	−0.65	0.12	2.01
World region (Ref = Western Pacific)										
African	−0.14	0.65	−0.73	0.46	2.99	0.10	0.70	−0.40	0.59	3.20
Eastern Mediterranean	−0.24	0.41	−0.81	0.34	2.40	−0.02	0.93	−0.50	0.45	2.59
European	0.56	0.10	−0.11	1.22	1.78	0.40	0.15	−0.14	0.94	1.82
Americas	−0.51	0.11	−1.14	0.11	2.02	−0.52	0.05	−1.04	−0.01	2.15
Southeast Asia	0.06	0.81	−0.46	0.59	3.10	0.13	0.56	−0.30	0.56	3.26
Length of stay in the US (Ref = Less than 12 months)										
12–24 months	**−0.41**	**0.05**	**−0.81**	**−0.01**	1.49	−0.31	0.06	−0.64	0.02	1.57
25–60 months	−0.29	0.15	−0.68	0.11	1.65	−0.16	0.31	−0.48	0.16	1.70
More than 60 months	−0.32	0.22	−0.85	0.20	1.43	−0.19	0.38	−0.62	0.24	1.47
University (Ref = University of Alabama										
The University of Mississippi/Oxford	0.04	0.98	−0.29	0.26	1.11	0.12	0.34	−1.22	0.03	1.12
Social support						**0.61**	**<0.001**	**0.44**	**0.79**	**1.38**
Acculturative stress						−0.12	0.15	−0.27	0.04	1.21
Cultural integration						0.16	0.11	−0.04	0.36	1.56
Alcohol consumption						−0.01	0.74	−0.04	0.03	1.30

Ref = Reference level, Bolded means significant at *p* < 0.05.

## Data Availability

The data presented in this study are available on request from the corresponding author due to privacy concerns.

## Supplementary Materials

The following supporting information can be downloaded at: https://www.mdpi.com/article/10.3390/ijerph22020253/s1, File S1: International Students Survey.
