# Supplementary material for: Predictors of Academic Adjustment Among International Students in Rural Southern USA
Source: Int J Environ Res Public Health. 2025 Feb 11;22(2):253. doi: 10.3390/ijerph22020253 (PMC11855055; doi:10.3390/ijerph22020253)
Supplement: Supplementary file 1 [file ijerph-22-00253-s001.zip › ijerph-3395100-supplementary.pdf]

Supplementary Materials File S1

International Students Survey

1. I have read and understood the information sheet and I consent to participating in this study

○ Yes

○ No

2. Please answer the following question(s) to determine if you are eligible for the study. If you are not eligible, you will be skipped to the end of the survey.

   Are you 21 years of age or older?

○ Yes

○ No

3. Please answer the following question(s) to determine if you are eligible for the study. If you are not eligible, you will be skipped to the end of the survey.

   Are you an International Student?

○ Yes

○ No

Background Characteristics

4. What is your age in years?

____

5. What is your gender?

- ○ Male
- ○ Female
- ○ Non-binary / Third gender
- ○ Other

6. How long have you lived in the US?

- ○ Less than 12 months (< 1 year)
- ○ 12-24 months (1 year-2 years)
- ○ 25-60 months (>2 years-5 years)
- ○ More than 60 months (>5 years)

7. Relationship status?

- ○ Single
- ○ Dating
- ○ Married
- ○ Divorced
- ○ Other

8. What is your current academic level?

- ○ Graduate
- ○ Undergraduate

9. What is the primary source of funding for your studies at present?

- ○ Graduate teaching/research assistantship
- ○ Scholarship
- ○ Family funds
- ○ Other

10. Looking at the map below, what world region is your country of origin?

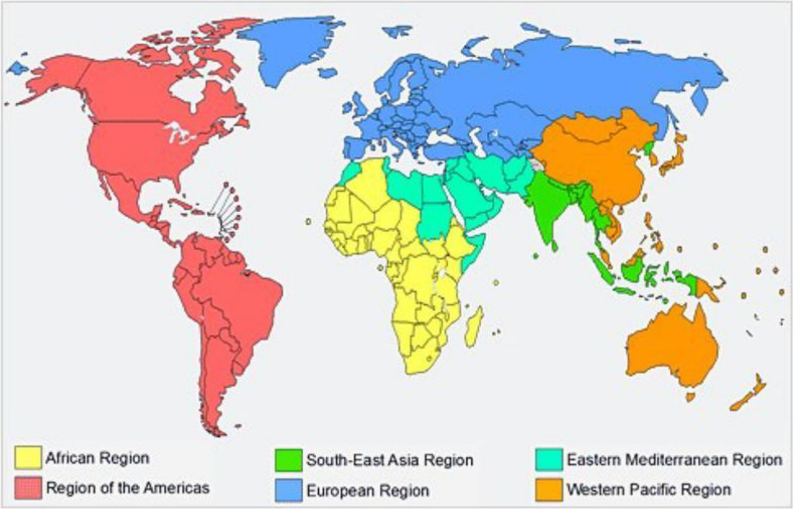

- ○ African Region
- ○ Region of the Americas
- ○ South-East Asia Region
- ○ European Region
- ○ Eastern Mediterranean Region
- ○ Western Pacific Region

Alcohol Use

11. How often do you have a drink containing alcohol?

- ◯ Never
- ◯ Monthly or less
- ◯ 2-4 times a month
- ◯ 2-3 times a week
- ◯ 4 or more times a week

12. How many units of alcohol do you drink on a typical day when you
are drinking?

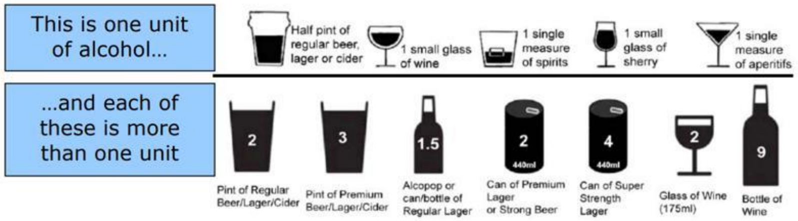

- ◯ 1-2

○ 3-4

○ 5-6

○ 7-9

○ 10 or more

13. How often have you had 6 or more units if female, or 8 or more if male, on a single occasion in the last year?

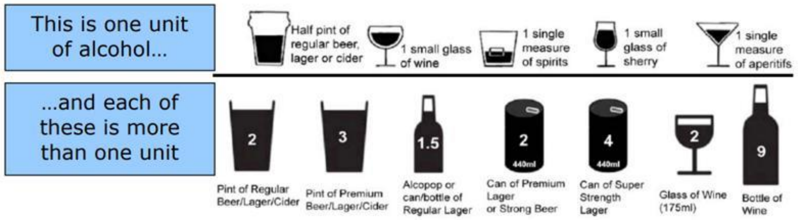

○ Never

○ Less than monthly

○ Monthly

○ Weekly

○ Daily or almost daily

14. How often during the last year have you found that you were not able to stop drinking once you had started?

Never

- [ ]

- [ ] Less than monthly

- [ ] Monthly

- [ ] Weekly

- [ ] Daily or almost daily

15. How often during the last year have you failed to do what was normally expected from you because of drinking?

- [ ] Never

- [ ] Less than monthly

- [ ] Monthly

- [ ] Weekly

- [ ] Daily or almost daily

16. How often during the last year have you needed a first drink in the morning to get yourself going after a heavy drinking session?

- [ ] Never

- [ ] Less than monthly

- [ ] Monthly

- ○ Weekly
- ○ Daily or almost daily

17. How often during the last year have you had a feeling of guilt or remorse after drinking?

- ○ Never
- ○ Less than monthly
- ○ Monthly
- ○ Weekly
- ○ Daily or almost daily

18. How often during the last year have you been unable to remember what happened the night before because you had been drinking?

- ○ Never
- ○ Less than monthly
- ○ Monthly
- ○ Weekly
- ○ Daily or almost daily

19. Have you or someone else been injured as a result of your drinking?

- No
- Yes, but not in the last year
- Yes, during the last year

20. Has a relative or friend, doctor or other health worker been concerned about your drinking or suggested that you cut down?

- No
- Yes, but not in the last year
- Yes, during the last year

Social Support

Please, read each statement about getting social support carefully. Please, rate how much you agree with each statement by clicking the corresponding options below.

21. My family really tries to help me.

- Strongly Disagree
- Somewhat Disagree
- Neutral
- Somewhat Agree

- Strongly Agree

22. I can get the emotional help and support I need from my family.

- Strongly Disagree
- Somewhat Disagree
- Neutral
- Somewhat Agree
- Strongly Agree

23. I can talk about my problems with my family.

- Strongly Disagree
- Somewhat Disagree
- Neutral
- Somewhat Agree
- Strongly Agree

24. My family is willing to help me make decisions.

- Strongly Disagree
- Somewhat Disagree

- Neutral
- Somewhat Agree
- Strongly Agree

25. I have friends with whom I can share my joys and sorrows.

- Strongly Disagree
- Somewhat Disagree
- Neutral
- Somewhat Agree
- Strongly Agree

26. My friends really try to help me.

- Strongly Disagree
- Somewhat Disagree
- Neutral
- Somewhat Agree
- Strongly Agree

27. I can talk about problems with my friends.

- Strongly Disagree
- Somewhat Disagree
- Neutral
- Somewhat Agree
- Strongly Agree

28. I can count on my friends when things go wrong.

- Strongly Disagree
- Somewhat Disagree
- Neutral
- Somewhat Agree
- Strongly Agree

29. There is a special person who is around when I am in need.

- Strongly Disagree

- Somewhat Disagree
- Neutral
- Somewhat Agree
- Strongly Agree

30. The International Students Center on campus is available when I need it.

- Strongly Disagree
- Somewhat Disagree
- Neutral
- Somewhat Agree
- Strongly Agree

31. I can talk about my problems with members in international students' organization.

- ○ Strongly Disagree
- ○ Somewhat Disagree
- ○ Neutral
- ○ Somewhat Agree
- ○ Strongly Agree

32. I trust university would offer me help when I need it.

- ○ Strongly Disagree
- ○ Somewhat Disagree
- ○ Neutral
- ○ Somewhat Agree
- ○ Strongly Agree

Acculturative stress

33. Homesickness bothers me.

- Strongly Disagree
- Somewhat Disagree
- Neutral
- Somewhat Agree
- Strongly Agree

34. I feel sad living in unfamiliar surroundings.

- Strongly Disagree
- Somewhat Disagree
- Neutral
- Somewhat Agree
- Strongly Agree

35. I miss the people and country of my origin.

- Strongly Disagree

- Somewhat Disagree
- Neutral
- Somewhat Agree
- Strongly Agree

36. I feel sad leaving my relatives behind

- Strongly Disagree
- Somewhat Disagree
- Neutral
- Somewhat Agree
- Strongly Agree

37. People show hatred toward me nonverbally.

- Strongly Disagree
- Somewhat Disagree
- Neutral
- Somewhat Agree

- Strongly Agree

38. People show hatred toward me verbally.

- Strongly Disagree
- Somewhat Disagree
- Neutral
- Somewhat Agree
- Strongly Agree

39. People show hatred me through actions

- Strongly Disagree
- Somewhat Disagree
- Neutral
- Somewhat Agree
- Strongly Agree

40. Others are sarcastic toward my cultural values.

- Strongly Disagree

- Somewhat Disagree
- Neutral
- Somewhat Agree
- Strongly Agree

41. Others don’t appreciate my cultural values

- Strongly Disagree
- Somewhat Disagree
- Neutral
- Somewhat Agree
- Strongly Agree

42. I fear for my personal safety because of my different cultural background.

- Strongly Disagree
- Somewhat Disagree
- Neutral
- Somewhat Agree

- Strongly Agree

43. I feel insecure here.

- Strongly Disagree
- Somewhat Disagree
- Neutral
- Somewhat Agree
- Strongly Agree

44. I frequently relocate for fear of others.

- Strongly Disagree
- Somewhat Disagree
- Neutral
- Somewhat Agree
- Strongly Agree

45. I generally keep a low profile due to fear.

- Strongly Disagree
- Somewhat Disagree
- Neutral
- Somewhat Agree
- Strongly Agree

46. I feel uncomfortable to adjust to new foods

- Strongly Disagree
- Somewhat Disagree
- Neutral
- Somewhat Agree
- Strongly Agree

47. Multiple pressures are placed on me after migration

- Strongly Disagree

- Somewhat Disagree
- Neutral
- Somewhat Agree
- Strongly Agree

48. I feel uncomfortable to adjust to new cultural values

- Strongly Disagree
- Somewhat Disagree
- Neutral
- Somewhat Agree
- Strongly Agree

49. I feel guilty to leave my family and friends behind

- ◯ Strongly Disagree
- ◯ Somewhat Disagree
- ◯ Neutral
- ◯ Somewhat Agree
- ◯ Strongly Agree

50. I feel guilty that I am living a different lifestyle here

- ◯ Strongly Disagree
- ◯ Somewhat Disagree
- ◯ Neutral
- ◯ Somewhat Agree
- ◯ Strongly Agree

51. I am treated differently in social situations

- Strongly Disagree
- Somewhat Disagree
- Neutral
- Somewhat Agree
- Strongly Agree

52. I feel that I receive unequal treatment

- Strongly Disagree
- Somewhat Disagree
- Neutral
- Somewhat Agree
- Strongly Agree

Acculturation

53. I often participate in mainstream American cultural traditions.

- Strongly Disagree
- Somewhat Disagree
- Neutral
- Somewhat Agree
- Strongly Agree

54. I would be willing to marry an American person.

- Strongly Disagree
- Somewhat Disagree
- Neutral
- Somewhat Agree
- Strongly Agree

55. I enjoy social activities with typical American people.

- ○ Strongly Disagree
- ○ Somewhat Disagree
- ○ Neutral
- ○ Somewhat Agree
- ○ Strongly Agree

56. I am comfortable interacting with typical American people.

- ○ Strongly Disagree
- ○ Somewhat Disagree
- ○ Neutral
- ○ Somewhat Agree
- ○ Strongly Agree

57. I enjoy American entertainment (e.g. movies, music).

- ○ Strongly Disagree
- ○ Somewhat Disagree

- Neutral
- Somewhat Agree
- Strongly Agree

58. I often behave in ways that are typically American.

- Strongly Disagree
- Somewhat Disagree
- Neutral
- Somewhat Agree
- Strongly Agree

59. It is important for me to maintain or develop American cultural practices.

- Strongly Disagree
- Somewhat Disagree
- Neutral
- Somewhat Agree
- Strongly Agree

60. I believe in mainstream American values.

- Strongly Disagree
- Somewhat Disagree
- Neutral
- Somewhat Agree
- Strongly Agree

61. I enjoy American jokes and humor.

- Strongly Disagree
- Somewhat Disagree
- Neutral
- Somewhat Agree
- Strongly Agree

62. I am interested in having American friends.

- Strongly Disagree
- Somewhat Disagree

- Neutral
- Somewhat Agree
- Strongly Agree

Academic Adaptation

63. I am satisfied with my academic progress

- Strongly Disagree
- Somewhat Disagree
- Neutral
- Somewhat Agree
- Strongly Agree

64. My teachers provide the necessary support when I need.

- Strongly Disagree
- Somewhat Disagree
- Neutral
- Somewhat Agree

- Strongly Agree

65. When I need help, my classmates are there for me.

- Strongly Disagree
- Somewhat Disagree
- Neutral
- Somewhat Agree
- Strongly Agree

66. I am comfortable with the teaching styles of my new teachers.

- Strongly Disagree
- Somewhat Disagree
- Neutral
- Somewhat Agree
- Strongly Agree

67. I collaborate with my classmates on school projects.

- Strongly Disagree
- Somewhat Disagree
- Neutral
- Somewhat Agree
- Strongly Agree

68. I feel supported by my university.

- Strongly Disagree
- Somewhat Disagree
- Neutral
- Somewhat Agree
- Strongly Agree
